# Teratoma to Angiosarcoma: A Metamorphosis in the Mediastinum

**DOI:** 10.7759/cureus.62555

**Published:** 2024-06-17

**Authors:** Sudeep Acharya, Ngowari Pokima, Ekrem Yetiskul, Michel Achkar, Yisroel Y Grabie, Sameer Khanijo, Manuel Villa Sanchez, Michel Chalhoub

**Affiliations:** 1 Pulmonary and Critical Care Medicine, Staten Island University Hospital, Staten Island, USA; 2 Internal Medicine, Staten Island University Hospital, Staten Isand, USA; 3 Internal Medicine, Staten Island University Hospital, Staten Island, USA; 4 Pulmonary and Critical Care Medicine, Donald and Barbara Zucker School of Medicine at Hofstra/Northwell/North Shore University Hospital and Long Island Jewish Medical Center, Manhasset, USA; 5 Thoracic Surgery, Staten Island University Hospital, Staten Island, USA

**Keywords:** clinical case report, tumor transformation, immature teratoma, mediastinum malignancy, germ cell tumors, high-grade angiosarcoma

## Abstract

We describe a rare and remarkable transformation of an immature mediastinal teratoma into high-grade angiosarcoma in a 21-year-old male. Mediastinal teratomas, particularly immature ones, are exceedingly rare, representing a small fraction of germ cell tumors (GCTs). Our case describes the clinical journey of the patient, who initially presented with acute chest pain and was subsequently diagnosed with an immature teratoma following imaging studies and elevated tumor markers. Despite an initial positive response to cisplatin-based chemotherapy, surveillance imaging revealed liver masses, which a biopsy confirmed as angiosarcoma. This transformation underscores the aggressive nature of immature teratomas and the propensity for sarcomatous differentiation, particularly in the mediastinum. The case contributes valuable insight into the management and surveillance of mediastinal non-seminoma germ cell tumors (MNGCT), a subset of GCTs with limited literature. We believe this case is the first in the literature to describe a transformation from an immature teratoma in the mediastinum to a high-grade angiosarcoma.

## Introduction

Extragonadal germ cell tumors (GCTs) are an uncommon group of tumors, constituting only 1% to 5% of all GCTs [1]. Within this subset, the mediastinum emerges as the predominant site for extragonadal GCTs, primarily afflicting males [2]. Notably, approximately 43% of mediastinal GCTs manifest as teratomas, comprising mature teratoma (63%), immature teratoma (4%), and teratoma with additional malignant components such as sarcoma, other malignant germ cell elements, or carcinoma (33%) [3]. 

An immature teratoma, also known as a malignant teratoma, represents a form of GCT typically composed of tissue from two or three germ cell layers: ectoderm, mesoderm, and endoderm [4,5]. An immature teratoma is an exceedingly rare tumor, accounting for only 1% of all teratomas [5]. Typically, these tumors are found in younger individuals, primarily during the first two decades of life, with the highest incidence observed between the ages of 15 and 19 [6]. A teratoma with malignant transformation (TMT) is a type of GCT that experiences a malignant change in its somatic teratomatous component, resulting in a histology that mirrors that of a somatic malignancy [7]. The malignant transformation of teratomas into the sarcoma component most commonly leads to the development of rhabdomyosarcoma, and angiosarcoma is the second most common [8]. Angiosarcoma, a rare malignant neoplasm originating from the vascular endothelium and representing only 1% of all soft tissue sarcomas (STS), can develop in any part of the body and is associated with a poor prognosis [8]. We report a case of a 21-year-old male who arrived at the emergency department with acute chest pain. Initial evaluations uncovered a large anterior mediastinal mass on imaging and elevated levels of alpha-fetoprotein (AFP) and beta-HCG. A biopsy confirmed the presence of an immature teratoma. Initially, the patient responded well to chemotherapy, but subsequent surveillance imaging revealed liver and spleen masses. A biopsy of the liver masses then revealed angiosarcoma.

## Case presentation

This case describes a 21-year-old male patient, previously in good health, who presented to our hospital with acute right substernal chest pain radiating to the flank and back associated with a dry cough and exertional dyspnea that started one month prior to presentation. He denied any fever, changes in bowel habits, weight loss, or weight gain. Family history is non-contributory. A physical exam revealed absent breath sounds in the right lung field with no wheezing, crackles, or rhonchi. The remainder of the physical exam was unremarkable.

**Figure 1:**
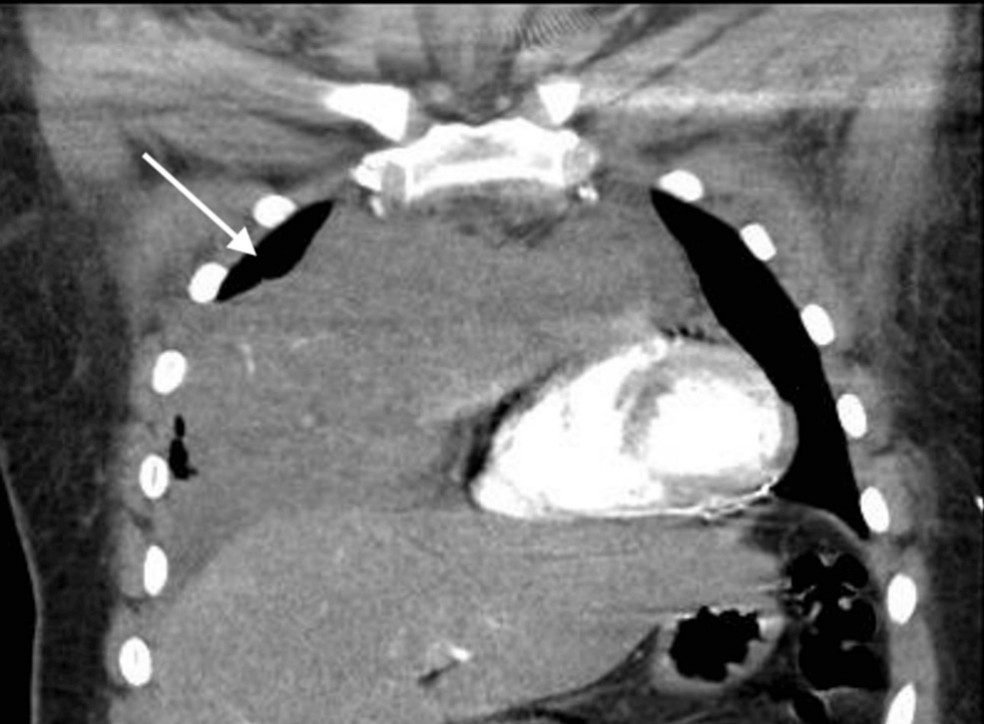
Computed tomography angiography of the chest. CT scan of the chest with coronal view showing the extent of the mediastinal mass and the mass effect on the heart. CT: computed tomography.

A CT scan of the chest revealed a large anterior mediastinal mass measuring 14.2 cm × 8.7 cm × 8 cm (Figure 1), exerting a mass effect on the right heart and superior vena cava.

**Figure 2:**
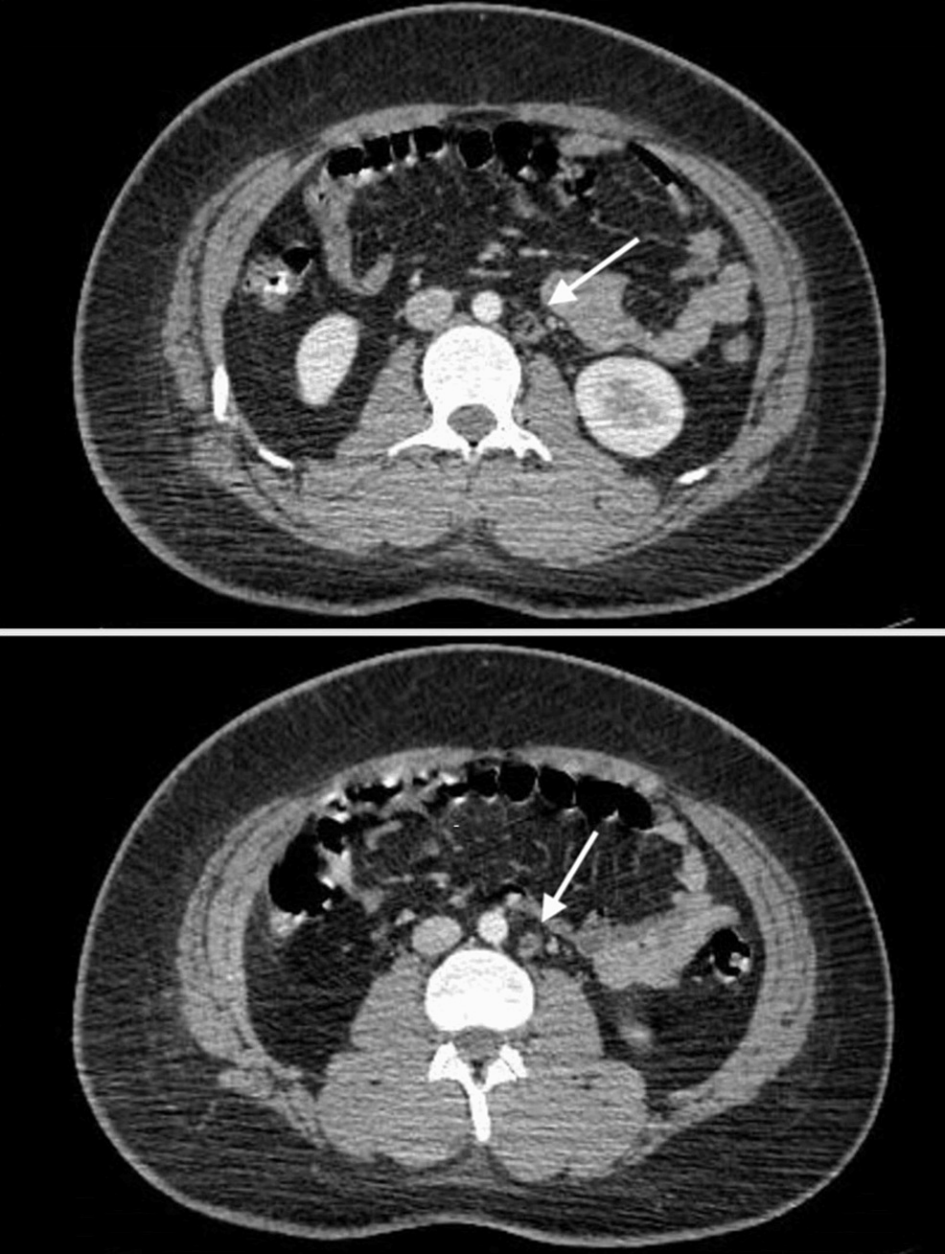
Computed tomography scan of the abdomen. Axial CT scan of the abdomen showing abdominal lymph node with fatty hilum.

The differential diagnosis at this initial stage included lymphoma, germ cell tumors, and thymic tumors. An ultrasound of the testis was conducted as part of the workup, showing a benign epididymal cyst of 0.4 cm. Additionally, a CT scan of the abdomen and pelvis revealed scattered retroperitoneal and abdominal lymph nodes, measuring up to 1.3 cm in the short axis, with a normal fatty hilum and reniform shape (Figure 2). No suspicious lesions or findings were noted in the liver, spleen, or any visceral organ.

Blood work showed an elevated lactate dehydrogenase (LDH) level of 251 U/L (reference range: 50-242 U/L), a human chorionic gonadotropin (HCG) level of 6 mUI/mL (reference range: <1 mUI/ml), and an AFP level of 3431 ng/ml (reference range: <8.3 mg/ml).

**Figure 3:**
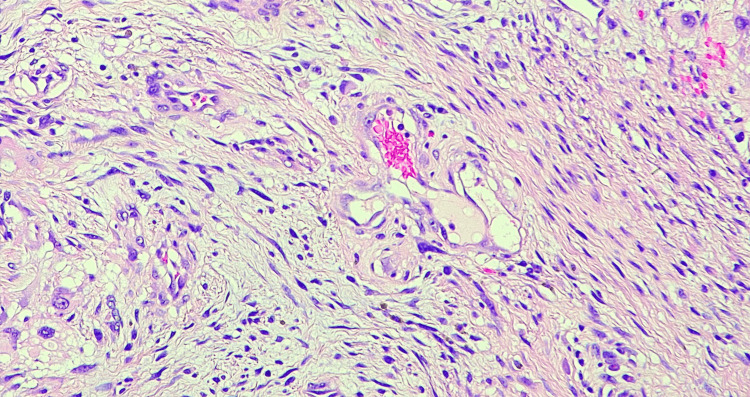
Pathology slide of the mediastinal mass. Pathology slide showing spindle cells with marked atypia and atypical mitosis.

A CT-guided biopsy of the mass was performed, leading to a diagnosis of spindle cell and malignant epithelioid tumor (Figure 3). The patient was started on neoadjuvent chemotherapy with a regimen of cisplatin, etoposide, and ifosfamide. During the patient's first cycle of chemotherapy, a cardiac echocardiogram identified a mobile echo structure in the right atrium, raising concerns about an atrial thrombus versus an atrial tumor. As a result, therapeutic anticoagulation was initiated. Repeat imaging of the chest and neck revealed compression of the superior vena cava by the tumor, causing superior vena cava syndrome. Prolonged and profound thrombocytopenia complicated the chemotherapy course.

**Figure 4:**
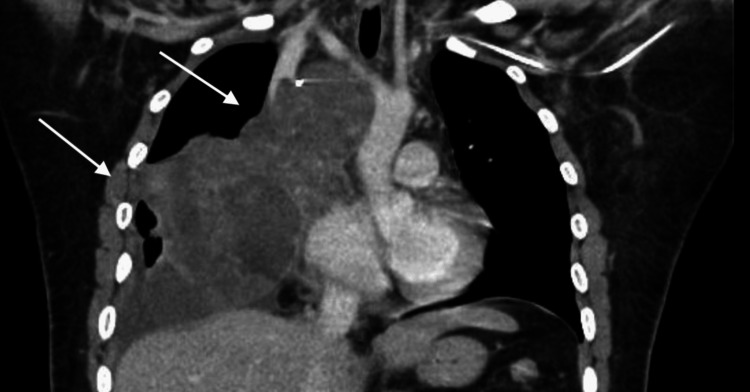
Computed tomography image of the chest after two cycles of chemotherapy. Coronal CT scan showing mediastinal mass (white arrows) compressing the heart and superior vena cava.

After completing the second cycle of chemotherapy, the patient visited the emergency department with facial edema. CT scans of the chest, abdomen, and pelvis showed an essentially unchanged thoracic mass exerting a mass effect on the heart (Figure 4).

**Figure 5:**
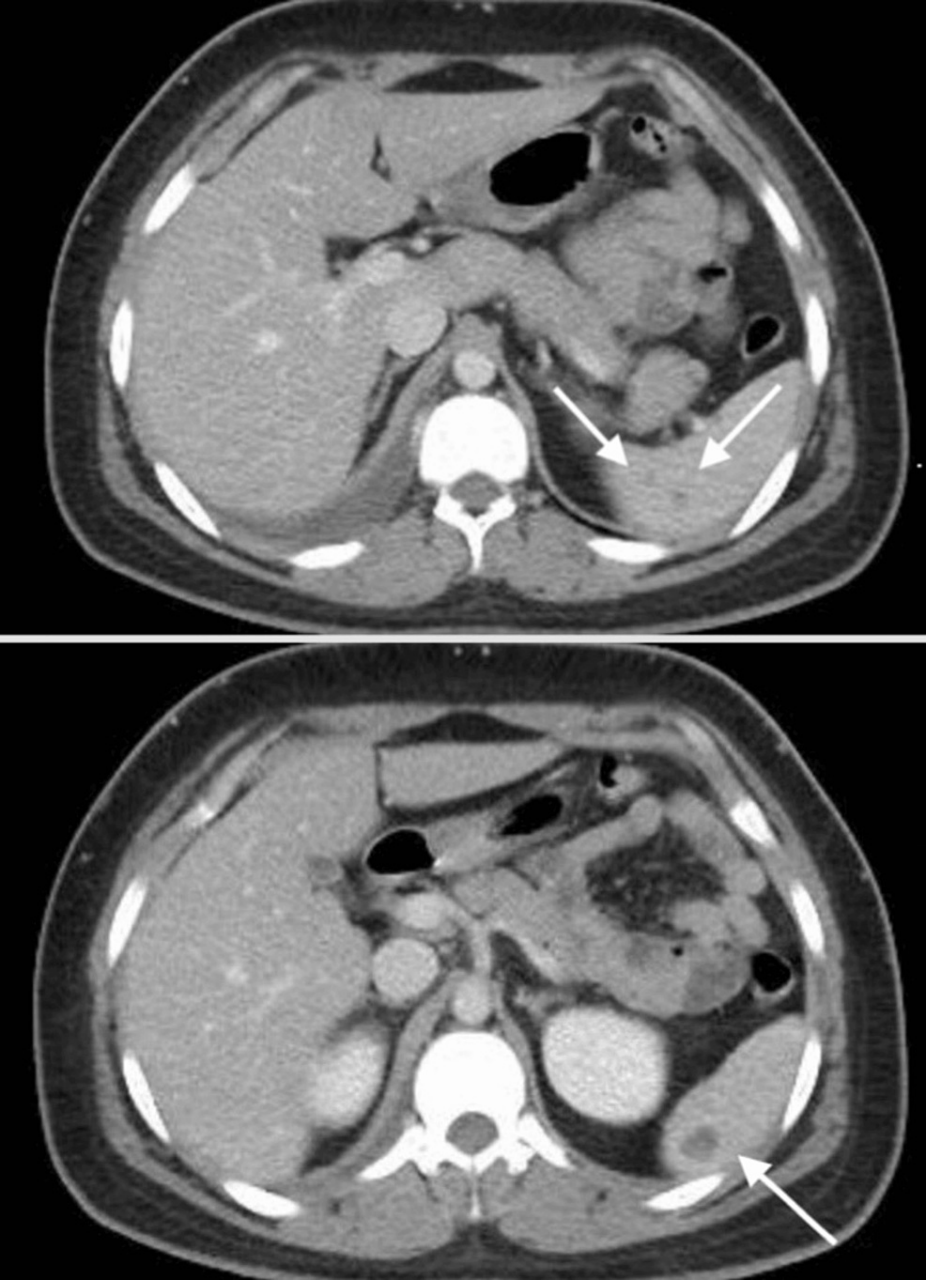
Computed tomography scan of the abdomen. Axial CT scan of the abdomen showing two splenic lesions (white arrows).

Two incompletely characterized splenic lesions measuring up to 1.2 cm were also found on the uppercuts of the abdomen (Figure 5).

Due to the poor response to chemotherapy, as evidenced by the same size of the tumor, the patient underwent a thoracotomy for the removal of the immature teratoma. The surgery revealed that the tumor was invading the pericardium, innominate vein, superior vena cava vein, and right phrenic nerve, necessitating a superior vena cava graft and diaphragm plication. The tumor was not found to be invading the diaphragmatic muscle. 

**Figure 6:**
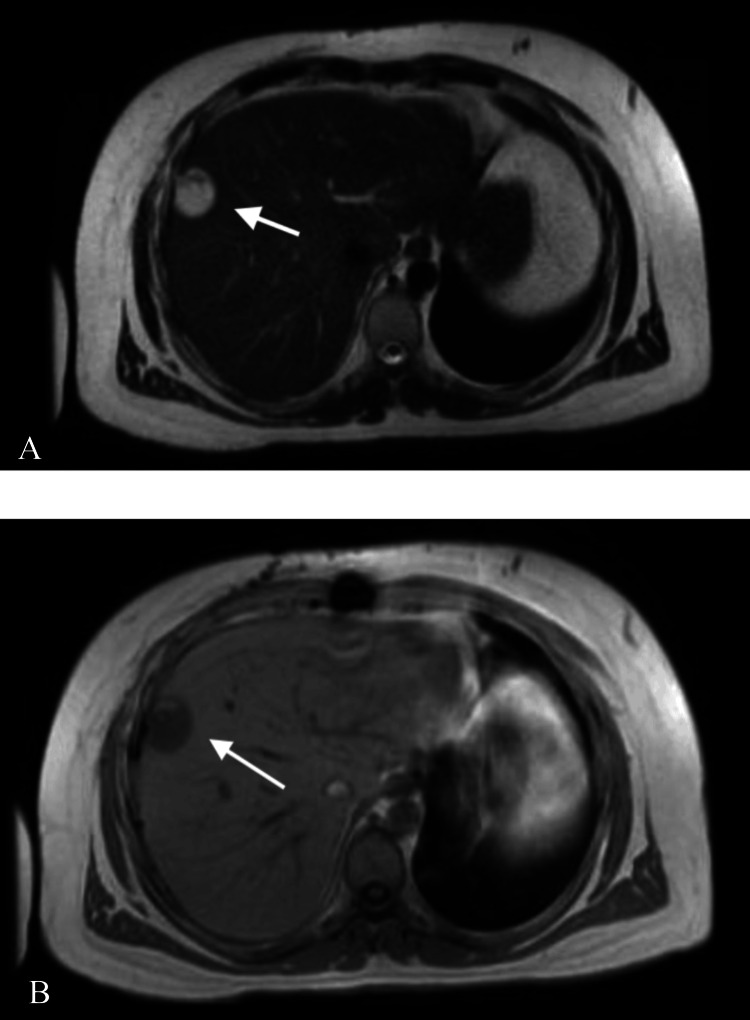
Magnetic resonance imaging of the liver. Axial MRI imaging of the abdomen [(A) T1 sequence and (B) T2 sequence] showing peripherally located liver mass.

**Figure 7:**
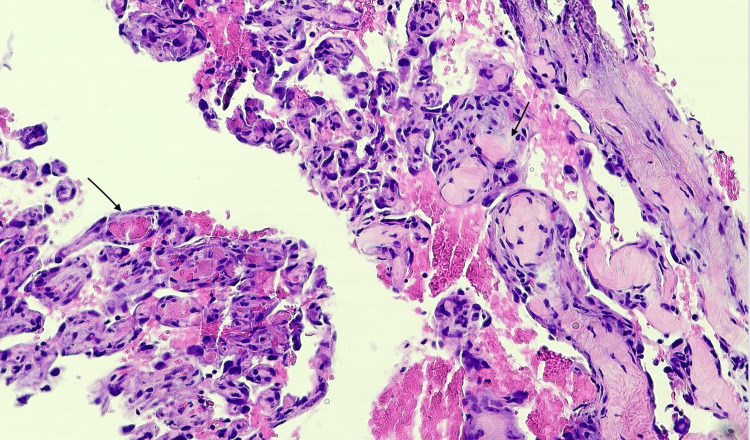
Pathology slide of the liver mass showing angiosarcoma. Anastomosing vascular channels lined by atypical endothelial cells (black arrows).

The patient was admitted to the cardio-thoracic unit in the postoperative period, where he achieved recovery. No acute postoperative complications were noted. One month after the excision, the patient required drainage of a pericardial effusion, at which time an MRI of the abdomen was performed to better assess the two splenic lesions. This MRI uncovered a liver lesion measuring 3.2 cm × 2.3 cm × 2.2 cm (Figure 6A-6B), along with multiple cystic/necrotic lesions in the spleen, raising concerns for metastasis. A biopsy of the liver lesion was performed, and the pathology report identified it as a high-grade angiosarcoma (Figure 7).

## Discussion

Our case highlights an intriguing case of a rare mediastinal immature teratoma transforming into an angiosarcoma in the liver. Limitations come from the fact that there are no further associations, in addition to the fact that the patient was lost to follow-up after having the biopsies for the liver mass.

In the adult mediastinum, the histological discrimination between pure seminoma and non-seminoma tumors (including teratoma, yolk sac tumor, choriocarcinoma, and embryonal tumors) is essential, as pure seminoma has a very favorable prognosis, often mirroring the benign clinical course of gonadal seminoma [1,9]. Due to the rarity of this tumor, comprehensive clinical data are scarce, with most data arising from case series. Data from studies with already small sample sizes is focused on non-seminoma germ cell tumors, a group of which immature teratomas make up a small subset. There remain uncertainties regarding the prognosis, optimal treatment, and surveillance practices of mediastinal non-seminoma germ cell tumors (MNGCT). This paper significantly contributes to addressing this knowledge gap within the field, casting light on an extremely rare subset of patients with limited existing literature.

The overall clinical outcome for MNGCT is worse than gonadal NGCTs [9]. Although optimal treatment is uncertain, immature teratoma is treated with four cycles of cisplatin-based chemotherapy followed by radical resection [10]. Favorable prognostic factors include complete resection, less than 10% residual viable tumor cells, and classification within the good prognosis group according to the International Germ Cell Consensus Classification Group (IGCCCG) [1,11]. All mediastinal non-seminoma GCTs are classified in the poor prognosis group [12].

Due to the rarity of the disease, the benefits of chemotherapy and the optimal timing of resection have yet to be established in randomized trials. Another potential course of treatment involves high-dose chemotherapy (HDCT) for patients with elevated postoperative serum tumor markers and recurrent non-seminoma germ cell tumors [13]. A multicenter trial compared HDCT to standard-dose chemotherapy (SDCT) in groups of patients classified as having a poor prognosis according to IGCCCG criteria. They found that two-year progression-free survival (75% vs. 59%) and overall survival (82% vs. 71%) were significantly prolonged in HDCT patients (P = 0.0056 and P = 0.0184, respectively) [14]. These results suggest that, within poor prognosis groups, HDCT should be considered superior to SDCT as first-line therapy. As heterogeneity exists within the poor prognosis group, additional studies will be necessary to refine the selection of patients likely to respond to this therapy.

Non-seminomatous mediastinal GCT has a poor prognosis with a five-year overall survival of 40-45% and poor response rates to chemotherapy, especially in recurrence [15]. These patients should be followed up closely at an experienced specialist center. Center experience in treating poor prognosis patients is associated with significantly better outcomes [3]. Follow-up should be every three months for the first two years and every six months for the next three years, with CT chest and tumor markers performed before each cycle of chemotherapy [3].

Sarcomatous differentiation of GCT, a phenomenon most commonly observed in the mediastinum, can occur in malignant GCTs, especially in teratoma [1]. This transformation may be present at the initial diagnosis but more commonly arises following chemotherapy or a late relapse, as seen in our patient. Rhabdomyosarcoma is the predominant type of differentiation, followed by angiosarcoma, leiomyosarcoma, and neuroblastoma [1,8]. Unfortunately, the prognosis for patients with a sarcomatous component is bleak [16]; these tumors are often unresponsive to cisplatin-based chemotherapy regimens [17]. Treatment should be modified to target the transformed histology, and surgical resection should be performed if possible [17].

## Conclusions

Although the differential diagnosis of mediastinal tumors is broad, malignant mediastinal teratoma should be considered. Treatment depends on the size of the tumor and symptoms at presentation but is usually multidisciplinary and involves surgery in addition to chemotherapy. Mediastinal malignant teratomas can present and evolve in a variety of ways. To date, we believe this to be the first case in the literature showing a transformation from immature teratomas in the mediastinum to high-grade angiosarcoma. This finding highlights the need for more active surveillance and screening strategies for this type of cancer.
